# Treatment of mantle cell lymphoma in Asia: a consensus paper from the Asian Lymphoma Study Group

**DOI:** 10.1186/s13045-020-00855-9

**Published:** 2020-03-17

**Authors:** Dok Hyun Yoon, Junning Cao, Tsai-Yun Chen, Koji Izutsu, Seok Jin Kim, Yok Lam Kwong, Tong Yu Lin, Lim Soon Thye, Bing Xu, Deok Hwan Yang, Won Seog Kim

**Affiliations:** 1 Asan Medical Center, University of Ulsan College of Medicine, Seoul, South Korea; 2 Fudan University Shanghai Cancer Center, Shanghai, China; 3 National Cheng Kung University Hospital, Tainan, Taiwan; 4 National Cancer Center Hospital, Tokyo, Japan; 5 School of Medicine, Sungkyunkwan University, Samsung Medical Center 115 Irown-Ro, Gangnam-Gu, Seoul, South Korea; 6 Queen Mary Hospital, Pok Fu Lam, Hong Kong; 7 Sun Yat-sen University Cancer Center, Guangzhou, China; 8 National Cancer Center, Singapore, Singapore; 9 Hospital of Xiamen University, Xiamen, China; 10 Chonnam National University Hwasun Hospital, Hwasun, South Korea

**Keywords:** Mantle cell lymphoma, Asia, Treatment, Guidelines

## Abstract

**Background:**

Mantle cell lymphoma (MCL) is a B cell malignancy that can be aggressive and with a poor prognosis; the clinical course is heterogeneous. The epidemiology of MCL in Asia is not well documented but appears to comprise 2–6% of all lymphoma cases based on available data, with variation observed between countries. Although international guidelines are available for the treatment of MCL, there is a lack of published data or guidance on the clinical characteristics and management of MCL in patient populations from Asia. This paper aims to review the available treatment and, where clinical gaps exist, provide expert consensus from the Asian Lymphoma Study Group (ALSG) on appropriate MCL management in Asia.

**Body:**

Management strategies for MCL are patient- and disease stage-specific and aim to achieve balance between efficacy outcomes and toxicity. For asymptomatic patients with clearly indolent disease, observation may be an appropriate strategy. For stage I/II disease, following international guidelines is appropriate, which include either a short course of conventional chemotherapy followed by consolidated radiotherapy, less aggressive chemotherapy regimens, or a combination of these approaches. For advanced disease, the approach is based on the age and fitness of the patient. For young, fit patients, the current practice for induction therapy differs across Asia, with cytarabine having an important role in this setting. Hematopoietic stem cell transplantation (HSCT) may be justified in selected patients because of the high relapse risk. In elderly patients, specific chemoimmunotherapy regimens available in each country/region are a treatment option. For maintenance therapy after first-line treatment, the choice of approach should be individualized, with cost being an important consideration within Asia. For relapsed/refractory disease, ibrutinib should be considered as well as other follow-on compounds, if available.

**Conclusion:**

Asian patient-specific data for the treatment of MCL are lacking, and the availability of treatment options differs between country/region within Asia. Therefore, there is no clear one-size-fits-all approach and further investigation on the most appropriate sequence of treatment that should be considered for this heterogeneous disease.

## Background

### MCL disease overview

Mantle cell lymphoma (MCL) is a rare B cell malignancy subtype and is typically clinically aggressive with a poor prognosis [1, 2]. The clinical course of MCL is heterogeneous, with some cases characterized by splenomegaly, peripheral blood lymphocytosis, and with little or no nodal disease, and thereby presenting with indolent features [3–7]. Patients with MCL may present with generalized lymphadenopathy; however, extra-nodal involvement is common in the peripheral blood, bone marrow, and gastrointestinal tract [8].

The diagnosis of MCL is based on lymph node biopsy, with histological review showing typical patterns including nodular, diffuse, pleomorphic, and blastoid—the latter two associated with particularly aggressive disease [9]. Typically, immunophenotyping shows CD19, CD20, CD22, CD43, CD79a, CD5, and FMC7 positivity and CD23, CD10, CD200, and BCL6 negativity. Evidence of t(11;14)(q13;q32) or cyclin D1 expression is required to diagnose MCL; however, approximately 5% of cases otherwise consistent with MCL may be cyclin D1-negative and may present with cyclin D2-positive disease instead [8]. Additional tests including SOX-11 should be considered in MCL to be distinguished from other diseases or to determine clinically indolent diseases [10]. Diagnostic workup also includes physical examination, viral serology (if rituximab treatment is contemplated), computed tomography (CT), positron emission tomography (PET)-CT or magnetic resonance imaging, and bone marrow aspiration and biopsy to enable the accurate staging of disease [11]. Cerebrospinal fluid evaluation with or without central nervous system prophylaxis should be performed in some cases [10]. Staging follows the Lugano classification (Table 1).

**Table 1:** Lugano classification for mantle cell lymphoma staging

Stage	Area of involvement
I	One lymph node region
IE	One extra-nodal site
II	Two or more lymph node regions
IIE	Localized extra-nodal sites on the same side of the diaphragm
III	Lymph node regions or lymphoid structures (e.g., thymus and Waldeyer’s ring) on both sides of the diaphragm
IV	Diffuse or disseminated extra-lymphatic organ involvement

### Review of MCL epidemiology in Asia

#### Epidemiological characteristics of MCL in Asia

The epidemiology of MCL in Asia is not well documented, with few published datasets describing the incidence and outcomes in Asian patients. Broadly, MCL accounts for 2 to 6% of total lymphoma cases, with variation seen between countries as described below.

In China, a review of 653 B cell lymphoma cases from a single center found MCL in 6.3% of patients [12]. Further data from Shanxi and Hubei recorded that MCL comprises 2.6% and 4% of total non-Hodgkin lymphoma (NHL) cases, respectively [13, 14]. Patients are generally diagnosed around 60 years of age and at stage III or IV [15, 16]. Similar patterns have been observed in patients from Hong Kong (3% of NHL cases) [17, 18]. In Taiwan, a single-center analysis found that MCL accounted for 4% of total malignant lymphoma and approximately 5% of non-Hodgkin B cell lymphoma cases [19]. In a retrospective analysis of 3998 patients in Korea, 2.4% of NHL cases were MCL, and data from the Korea Central Cancer Registry describe an age-standardized MCL incidence rate of 0.09 per 100,000, compared with 0.24 per 100,000 in Asian Americans [20, 21]. In Japan, MCL comprised 3.3% of all lymphoma cases [22]. In a review of 708 lymphoid malignancy cases, also in Japan, MCL was the fourth most common at 5.9% [23]. Finally, the Singapore Cancer Registry found MCL accounted for 1.2% of lymphoid neoplasms from 1998 to 2012 [24].

### Survival outcome of MCL in Asia

The reported survival rates of patients with MCL, at least in East Asia, were similar to those of Western countries. Five-year overall survival (OS) and progression-free survival (PFS) rates reported in China in 2017 were 35.5% and 8.8%, respectively [16]. Taiwan has recorded a relatively better prognosis, with 5-year survival rates of 78% in 2006 though a small number of patients were included in these analyses [19, 25, 26]. A Korean retrospective review of the medical records of 131 patients with MCL showed a similar pattern of presentation and prognosis as seen in mainland China, Hong Kong, and Taiwan, with 2-year OS and PFS of 64.7% and 39.7%, respectively [27].

### Lack of treatment guidance for MCL in Asian populations and need for developing this consensus statement

Treatment guidelines have been developed internationally, in particular, by the National Comprehensive Cancer Network (NCCN), the European Society of Medical Oncology (ESMO), and the British Society of Hematology (BSH) [7, 10, 11]. However, published data or guidance on the clinical characteristics and management of MCL are generally lacking for patient populations from Asia. Importantly, the level of healthcare resources and available treatments in Asia vary from one country/region to another. While some areas may have only basic access to oncology care, some highly urbanized areas may have world-class expertise and more novel agents at their disposal [28, 29]. Moreover, Asian patients may have different comorbidities, such as hepatitis B virus infection. Infection with hepatitis B virus is a common comorbidity in patients with lymphoma in Asia, with a 2- to 5-fold higher prevalence among patients with NHL compared with the general population [29]. In these patients who have inactive hepatitis B virus infection, chemotherapy may lead to hepatitis B virus reactivation, and thus, this should be an important consideration for the management of patients with MCL in Asia.

In this context, guidelines from Western regions may not be appropriate in Asia, and tailored treatment guidance may be needed.

This paper aims to review the available treatment options and, where clinical gaps exist, to provide expert consensus from the ALSG on the appropriate management of MCL in Asian patients.

## Consensus process

The consensus was developed by the Asia Pacific MCL Working Group of the ALSG. The Working Group was composed of 11 expert oncologists and hematologists from East Asia and Southeast Asia. A consensus meeting was convened in March 2019 in Incheon, Korea, wherein the Working Group reviewed and discussed the available evidence for the treatment of various subsets of patients with MCL in Asia. Consensus recommendations were formed based on the available clinical evidence and the collective experience of the Working Group members, with consideration for the various levels of healthcare resources present in their represented country/region. The recommendations were revised until full consensus was agreed among the Working Group members.

## Management of MCL: review of evidence, available guidelines, and ALSG expert opinion

Management strategies for MCL are patient- and disease stage-specific, aiming to achieve balance between efficacy outcomes and toxicity. Strategies not only must consider key patient considerations such as age and fitness, but also must account for younger patients’ desire to preserve fertility.

This section briefly reviews available treatments and guidance developed for managing MCL in various disease stages and patient subtypes by key international organizations (NCCN, ESMO, and BSH) reviews current supporting data from Asia and summarizes the ALSG recommendations.

### Overview of currently available therapies for MCL

Various therapeutics are used in the management of MCL, with different mechanisms of action and targets that allow the use of a wide variety of combination regimens. Traditional chemotherapeutic agents used include cyclophosphamide, doxorubicin, vincristine, cytarabine (Ara-C), cisplatin, and bendamustine. These are often combined in chemotherapeutic regimens with a steroid. Recently, the monoclonal antibody, rituximab; the immunomodulatory agent, lenalidomide; and the proteasome inhibitor, bortezomib, have been incorporated into treatment regimens, followed by the introduction of newer, small-molecule inhibitors (i.e., ibrutinib and acalabrutinib). Finally, a role remains for autologous hematopoietic stem cell transplant (auto-HSCT) and allogeneic hematopoietic stem cell transplant (allo-HSCT) in MCL. While conventional chemotherapy plays a vital role in the treatment of MCL, there is still limited robust evidence to support its use. A summary of available therapies is shown in Table 2.

**Table 2:** Key medications used globally in the management of MCL

Agent	Class	Availability in Asia
Cyclophosphamide	Alkylating agent	Widely available
Doxorubicin	Anthracycline	Widely available
Vincristine	Microtubule assembly blocker	Widely available
Cytarabine	DNA polymerase inhibitor	Cytarabine is the backbone of chemotherapy regimens in Asia
Cisplatin	DNA replication inhibitor	Widely available
Lenalidomide	Immunomodulatory agent	Approved and reimbursed in limited countries
Bendamustine	Alkylating agent	Approved and reimbursed excluding Korea
Rituximab	Monoclonal antibody	Approved and reimbursed as R-CHOP or R-HyperCVAD R-maintenance is limitedly available
Temsirolimus	mTOR inhibitor	Rarely used outside of Asia
Bortezomib	Proteasome inhibitor	Approved and reimbursed for first-line use as VR-CAP
Acalabrutinib	BTK inhibitor	Not available in Asia
Zanubrutinib	BTK inhibitor	Not available in Asia
Ibrutinib	BTK inhibitor	Widely approved and reimbursed for rrMCL
Venetoclax	BH3-mimetic	Approved for MCL in limited countries

*BH3* B cell lymphoma 2 homology 3; *BTK* Bruton’s tyrosine kinase; *MCL* mantle cell lymphoma; *mTOR* mammalian target of rapamycin; *R-CHOP* rituximab, cyclophosphamide, doxorubicin, vincristine, prednisolone; *R-hyperVCAD* rituximab, hyperfractionated cyclophosphamide, vincristine, doxorubicin, dexamethasone + methotrexate and high-dose cytarabine; *rrMCL* relapsed/refractory mantle cell lymphoma;*VR-CAP* bortezomib, rituximab, cyclophosphamide, doxorubicin, prednisone

### First-line treatment

#### Indolent MCL

The typical clinical presentation of indolent disease comprises leukemic non-nodal CLL-like, including splenomegaly, low tumor burden, and Ki-67 proliferation fraction < 10% [10]. It is not clear if earlier treatment of younger, asymptomatic patients with indolent MCL offers any advantage [7]. Guidelines generally recommend a watch-and-wait approach for indolent MCL, generally in SOX11-negative disease and in patients who are otherwise well [7, 10, 11]. Data from Weill Cornell Medical Center suggest to use a close observation strategy with deferred therapy in selected asymptomatic patients with newly diagnosed MCL, which showed a longer survival in the observational group versus the early treatment group [6]. Real-world observational data from the Nordic Lymphoma Group demonstrated no difference in OS among patients managed with a watch-and-wait strategy versus radiotherapy [30].

For patients with indolent MCL who are developing symptoms or have any other indication for treatment, NCCN guidelines recommend re-biopsy and TP53 mutation testing to predict the treatment course [10]. TP53 negativity and treatment naivety indicate the need for aggressive management. Conversely, the appropriate treatment course for patients with TP53-positive disease is unknown, and chemotherapy in TP53-mutated disease is generally less effective. Therefore, while conventional chemotherapy, including transplantation, could be used in this patient population, enrollment in a clinical trial is strongly suggested where possible.

No clinical data are available describing the management of or intervention for Asian patients with indolent MCL.

**Table:** 

**ALSG consensus for indolent MCL** Adoption of a management strategy similar to CLL, utilizing a “watch-and-wait” approach, may be appropriate for asymptomatic patients with MCL. Typical clinical presentation of indolent disease comprises leukemic non-nodal CLL-like, including splenomegaly, low tumor burden, and Ki-67 proliferation fraction < 10%; it is useful to confirm SOX11 negativity with hypermutated IGHV to determine clearly indolent disease. Notably, patients may be reluctant to undertake a watch-and-wait strategy. For asymptomatic patients desiring treatment, the same treatment scheme for symptomatic patients requiring treatment is considered. Communication between the clinician and the patient, as well as caregivers, in the decision-making process is recommended. Clinical trial enrollment is strongly suggested where possible.	

#### Stage I/II limited, non-bulky disease

MCL is usually diagnosed at an advanced stage, and stage I/II MCL is rare [31, 32] and some of these patients (up to 50%) have gastrointestinal involvement [32], which may be detected on gastroscopy and colonoscopy [31]. For patients with stage I/II limited, non-bulky disease, ESMO guidelines recommend a short course of conventional chemotherapy induction followed by consolidated radiotherapy [11]. This is based on the conflicting data describing both long-term remission and relapse within 1 year following radiotherapy. For this population, NCCN recommends either radiotherapy, chemotherapy with less aggressive regimens, or a combination of the two [10]. Depending on patient response to therapy, the next steps could be observation every 3–6 months or proceeding to more aggressive treatments.

**Table:** 

**ALSG consensus for stage I/II limited, non-bulky disease** Following ESMO and NCCN guidelines is appropriate for Asian patients with MCL. However, consideration could also be made to treat according to guideline recommendations for advanced disease, particularly for patients with adverse histological features.	

#### Advanced stage disease

Once treatment is required, the choice of regimen is based on age, the presence of comorbidities, performance status, and the goal of therapy. Patients are categorized as “young fit” and suitable for auto-HSCT, or “elderly unfit,” where HSCT is not appropriate [7].

##### Advanced stage: young fit patients

International treatment guidelines recommend various intensive therapies as induction therapy for HSCT in young fit patients, with cytarabine-containing regimens predominately forming the backbone of therapy [7, 11, 33, 34]. For patients who are candidates for high-dose therapy and/or HSCT, NCCN recommends aggressive induction therapy with R-DHAP (rituximab, dexamethasone, cytarabine, cisplatin), alternating R-CHOP (rituximab, cyclophosphamide, doxorubicin, vincristine, prednisolone)/R-DHAP, the Nordic regimen (i.e., rituximab with alternating cycles of cyclophosphamide, doxorubicin, vincristine, prednisone [Maxi-CHOP], and high-dose cytarabine), or R-HyperCVAD (hyperfractionated cyclophosphamide, vincristine, doxorubicin, dexamethasone, alternating with high-dose methotrexate and cytarabine) [10]. BR could also be considered.

HyperCVAD showed impressive complete response (CR) rates in a single-center study [35]. Rituximab combined with HyperCVAD (i.e., R-HyperCVAD) also has high CR rates, but up to 40% of patients are unable to complete the planned treatment; therefore, it is not recommended as first-line therapy by the BSH [7]. The Nordic MCL2 protocol of R-maxi-CHOP/high-dose cytarabine demonstrated event-free survival (EFS) of over 60% at 5 years [36]. Alternating R-CHOP/R-DHAP induction before HSCT has demonstrated a 5-year OS of 75% [37], and the use of high-dose cytarabine following alternating R-CHOP/R-DHAP offered additional clinical benefit showing a time to treatment failure of 65% and OS of 76% at 5 years [38].

Phase II clinical trial results suggest the use of BR followed by rituximab with high-dose cytarabine is generally well tolerated, with a 13-month PFS of 96% [39]. Additionally, the outcome of BR from the S1106 study is consistent, with comparable efficacy (2-year PFS rate of 81%) and an acceptable safety profile versus R-hyperCVAD reported [40].

Particular notice should be given to patients with hepatitis B virus infection—a common comorbidity in Asian patient populations. Close viral load monitoring and prophylactic antiviral medication are recommended [11, 41].

Very few datasets have been published on the management of MCL in young, fit patients in Asia. A retrospective study of 64 newly diagnosed MCL patients by the Lymphoma Treatment Study Group in Japan aimed to investigate the benefits of more intensive therapy and stem cell transplantation, but the study did not identify any benefit for specific regimens as induction therapy [42].

**Table:** 

**ALSG consensus for advanced stage MCL in young, fit patients** Current practice for induction therapy in young, fit patients differs across Asia based on drug availability and reimbursement status. Few physicians use HyperCVAD; however, this approach risks omitting or reducing the dose of cytarabine, which plays an important role in induction therapy and may be the most useful drug. Cytarabine has an important role in induction therapy in MCL, but there is a lack of data supporting the best cytarabine dosage and dosing interval. The interaction between cytarabine and purine analogs should be considered to address concerns around cytarabine dose and toxicity. Clinical trial enrollment is strongly suggested where possible, especially for patients with TP53 mutation, which is associated with poor prognosis by conventional treatment.	

##### Advanced stage: elderly, unfit patients

International guidelines generally recommend that elderly, unfit patients who do not qualify for aggressive therapy should receive first-line treatment with less intensive conventional chemotherapy in combination with rituximab (e.g., R-CHOP, VR-CAP [bortezomib, rituximab, cyclophosphamide, doxorubicin, prednisone], BR, R-BAC [rituximab, cytarabine, bendamustine], modified R-HyperCVAD, or lenalidomide in combination with rituximab) [7, 10, 11]. The addition of rituximab to conventional chemotherapy improves response rates, PFS, and OS [43]. R-CHOP was shown to be superior to CHOP (objective response rate [ORR] 94% versus 75%) or R-FC (rituximab, fludarabine, cyclophosphamide; ORR 86% versus 78%) [44, 45]. Two further clinical trials showed VR-CAP and BR were superior to R-CHOP (ORR 92% versus 89% and 93% versus 91%, respectively) [46, 47]. Targeted therapy (including lenalidomide and rituximab) may be considered based on the need for a lower toxicity profile; however, rituximab monotherapy is not recommended based on inadequate response rates [11, 48, 49].

A multi-country study of bortezomib-based VR-CAP therapy for transplant-ineligible East Asian patients with newly diagnosed MCL was conducted in 121 patients from China, Taiwan, Japan, and Korea [50]. PFS was superior with VR-CAP versus R-CHOP recipients at 42 months follow-up, with a 43% improvement (28.6 versus 13.9 months). However, VR-CAP was associated with increased toxicity compared with R-CHOP in this population. A retrospective analysis of 131 Korean patients with MCL treated between 2004 and 2009 found that rituximab-containing regimens were used in over half of patients, with R-CHOP being the most frequent, used in 41.2% of patients; the OS and EFS at 2 years was 64.7% and 39.7%, respectively [27]. A study from Japan investigated the use of BR for previously untreated patients with indolent B cell NHL or MCL [51]. Among the 10 patients with MCL included in the analysis, 50% had CR and the estimated PFS at 30 months was 67.5% (95% CI 20.1–88.2). No treatment-related deaths were reported; however, major grade 3/4 toxicities included lymphopenia (97%), CD4 lymphopenia (91%), neutropenia (86%), and leukopenia (83%). Of note, bendamustine is associated with a reduction in CD4 lymphocyte counts and an increased risk of opportunistic infections [52].

**Table:** 

**ALSG consensus for advanced stage MCL in elderly, unfit patients** The choice of therapy in Asia is limited by drug availability and reimbursement status. R-CHOP, R-BAC, CHOP, and VR-CAP are used in this setting in Asia, while BR is the preferred regimen in elderly, unfit patients. Bendamustine is associated with lower CD4 counts and an increased risk of infection. These risks must be monitored closely when considering this agent and prophylaxis for pneumocystis pneumonia should be considered. Clinical trial enrollment is strongly suggested where possible, especially for patients with TP53 mutation, which is associated with poor prognosis by conventional treatment.	

##### Role of hematopoietic stem cell transplantation

International treatment guidelines stratify patients into suitability for ASCT and build treatment strategies around this, emphasizing the importance of ASCT in MCL management. However, limited data support this strategy, with only one prospective study comparing ASCT with non-transplant strategies. This study showed a PFS advantage with high-dose therapy and ASCT versus CHOP induction and interferon; however, no OS benefit was seen [33]. Additionally, an analysis of patients with high Mantle Cell Lymphoma International Prognostic Index (MIPI) scores showed poor outcomes despite receipt of HSCT [53, 54]. The use of ASCT may offer the chance to significantly prolong the duration of response, and most benefits are seen in patients who achieve complete remission [7].

ESMO guidelines highlight the lack of current data to support allo-SCT as first-line therapy [11, 55]. NCCN guidelines reserve allo-SCT, either myeloablative or nonmyeloablative, for second-line consolidation therapy [10]. Some data have suggested a prolonged PFS in patients receiving allo-SCT after prior SCT or in refractory disease; however, only a minority of such patients can be expected to be cured [7, 56, 57].

Further data from the Japan Lymphoma Treatment Study Group’s retrospective review of MCL cases demonstrated a benefit of high-dose chemotherapy plus HSCT upfront or as salvage therapy versus no HSCT [42]. These data support the global experience on which the international guidelines are formed.

**Table:** 

**ALSG consensus on the role of hematopoietic stem cell transplantation** Some ALSG members felt that the high chance of relapse within 3–4 years, even with consolidation therapy, may justify the use of ASCT in young, fit patients. While the recommendation that all responding patients should receive ASCT has become generally accepted, this remains uncertain, especially with novel agents such as the Bruton’s tyrosine kinase (BTK) inhibitor ibrutinib changing the therapeutic landscape. Therefore, for young women of reproductive age, ASCT could be avoided and in relatively older individuals for whom reproduction is not a concern, ASCT remains an important option. In most countries in Asia, HSCT is reimbursed by payors whereas novel therapies are not. The decision for transplant versus alternative management strategies may be financially driven rather than data-driven. Real-world data may demonstrate the role or benefit of auto-SCT in some patients. Whether high-dose therapy followed by ASCT can be safely omitted from intensive first-line therapy that incorporates a BTK inhibitor will be tested in the European MCL Network TRIANGLE trial (NCT02858258).	

##### Maintenance therapy after first-line treatment

Limited data are available describing the benefit of maintenance therapy following first-line treatment. Rituximab has been the most frequently studied maintenance therapy and has been shown to improve both PFS and OS after R-CHOP therapy versus interferon in the European MCL Network study [45]. Preliminary data suggest a role for rituximab maintenance after ASCT in younger patients, with better PFS and OS reported [8, 58, 59]. Furthermore, the phase III Mantle Cell Lymphoma Efficacy of Rituximab Maintenance (LyMA) study assessed patients with treatment-naive MCL who received R-DHAP induction and ASCT followed by a random assignment to rituximab maintenance or observation [8, 34]. Patients randomly assigned to receive rituximab had superior EFS at 3 years compared with those randomized to observation (88.1% versus 73.4%). Rituximab plus radio-immunotherapy consolidation was also shown to offer a smaller PFS benefit following chemotherapy treatment [60].

A 2018 meta-analysis of rituximab-maintenance therapy strategies concluded that rituximab-maintenance therapy offers clinical benefit when used following R-CHOP or cytarabine-containing induction therapy for transplant-eligible patients and following R-CHOP in the relapse setting; however, the benefit after bendamustine- or fludarabine-containing induction therapies remains unclear [61].

International clinical practice guidelines differ in their recommendations for rituximab-maintenance therapy, with NCCN and BSH guidelines recommending its use following rituximab-containing chemotherapy regimens in patients not suitable for ASCT [7, 10].

There are no published data from Asian patient populations describing the efficacy of maintenance therapy after first-line treatment for MCL.

**Table:** 

**ALSG consensus for maintenance therapy after first-line treatment** The clinical benefit of rituximab-maintenance therapy following BR and VR-CAP is not convincing. Rituximab-maintenance therapy should be recommended after R-CHOP or cytarabine-containing induction therapy. The choice of rituximab-maintenance therapy in Asian patients should be individualized, and affordability and reimbursement status remain important considerations in the region.	

### Relapsed/refractory disease

Guidelines agree that the choice of salvage therapy is influenced by the prior lines of therapy used and duration of response to prior therapy. Non-cross-resistant regimens are preferential, specifically the use of newer targeted approaches for patients with early relapse, with the BTK inhibitor ibrutinib showing the highest response rates [11, 62–64]. If ibrutinib is contraindicated, lenalidomide with or without rituximab may offer some clinical benefit [11, 65–67].

Ibrutinib monotherapy demonstrated an ORR of 68%, a CR rate of 21%, and a median PFS of 13.9 months, with an additional benefit demonstrated with the addition of rituximab (ORR 87%; CR 38%; 15-month PFS 69%) [63, 64]. In the 3-year follow-up of the RAY study, ibrutinib showed a favorable OS trend versus temsirolimus (median OS 30.3 versus 23.5 months; hazard ratio [HR] 0.74 [95% CI 0.54–1.02], *P* = 0.0621), with the most benefit seen in patients receiving only one prior line of therapy [68]. In addition, in a pooled analysis after an extended 3.5-year follow-up of phase II and III clinical trials of patients with relapsed/refractory MCL, those who received second-line therapy and those achieving a CR derived the greatest benefit from ibrutinib treatment; median PFS and OS were 12.5 and 26.7 months, respectively [69].

The use of bortezomib-based chemotherapy showed an ORR of 33%, a CR rate of 8%, and a median PFS of 6.5 months; responses were improved with the addition of rituximab (ORR 58%; CR 16%) [70, 71]. Lenalidomide demonstrated limited clinical benefit with an ORR of 28%, a CR rate of 8%, and a median PFS of 4 months. These rates increased with the addition of rituximab to an ORR of 57%, a CR rate of 36%, and a median PFS of 11.1 months [66, 72]. Follow-on BTK inhibitors include acalabrutinib, which has demonstrated an ORR of 81%, a CR rate of 40%, and a 67% PFS at 12 months, and zanubrutinib, which has demonstrated an ORR of 86.5%, a CR rate of 29.7%, and a median PFS of 15.4 months [73, 74]. These findings should be confirmed in further studies with larger sample sizes.

Rituximab monotherapy in a small phase II study in Japanese patients with relapsed MCL demonstrated an ORR of 46% (6/13 patients) [75]. A subsequent analysis of factors affecting response in this study suggested that the ORR is higher in rituximab-treated patients receiving one versus two or more prior lines of chemotherapy, and PFS was shorter in patients with extra-nodal disease and those receiving two or more prior lines of chemotherapy [76]. Phase I/II clinical trial data demonstrated consistency in results seen for ibrutinib in Japanese patients, with a durable response over a median of 22.5 months [77, 78]. ORR was 93.8%, with CR seen in 31.3% of patients by the end of the phase II follow-up [78]. Real-world data of ibrutinib monotherapy in a salvage setting in Korea showed a favorable ORR and duration of response; however, higher MIPI and/or prior bendamustine exposure was associated with ibrutinib treatment failure and poorer outcomes [79]. Additional data from a Korean retrospective analysis of 75 patients with advanced MCL treated with ibrutinib reported a 70% ORR; at a median follow-up of 30.5 months, the median PFS was 18.6 months; and among elderly patients, better outcomes from ibrutinib were achieved in second-line versus later lines of therapy (median PFS 24.4 versus 5.8 months; *P* = 0.015) [80].

**Table:** 

**ALSG consensus for relapsed/refractory disease** Ibrutinib should be considered in the second-line rather than later-line setting. Ibrutinib is tolerable in Asian patients. Adverse events observed in clinical practice (e.g., non-specific musculoskeletal symptoms, skin dryness or itching, changes to nails and hair) are not well characterized in the literature and more Asian real-world data might be needed. Follow-on BTK inhibitors including zanubrutinib could be considered if available, and a difference to ibrutinib in terms of efficacy and safety remains to be shown. Clinical trial enrollment is strongly suggested where possible, especially for patients with TP53 mutation associated with poor prognosis.	

## Summary of MCL management in Asian patients

As reviewed in this paper, Asian patient-specific data for MCL epidemiology and treatment are lacking, and the availability of different treatment options makes the development of comprehensive MCL clinical practice guidelines for Asian patients challenging.

**Fig. 1:**
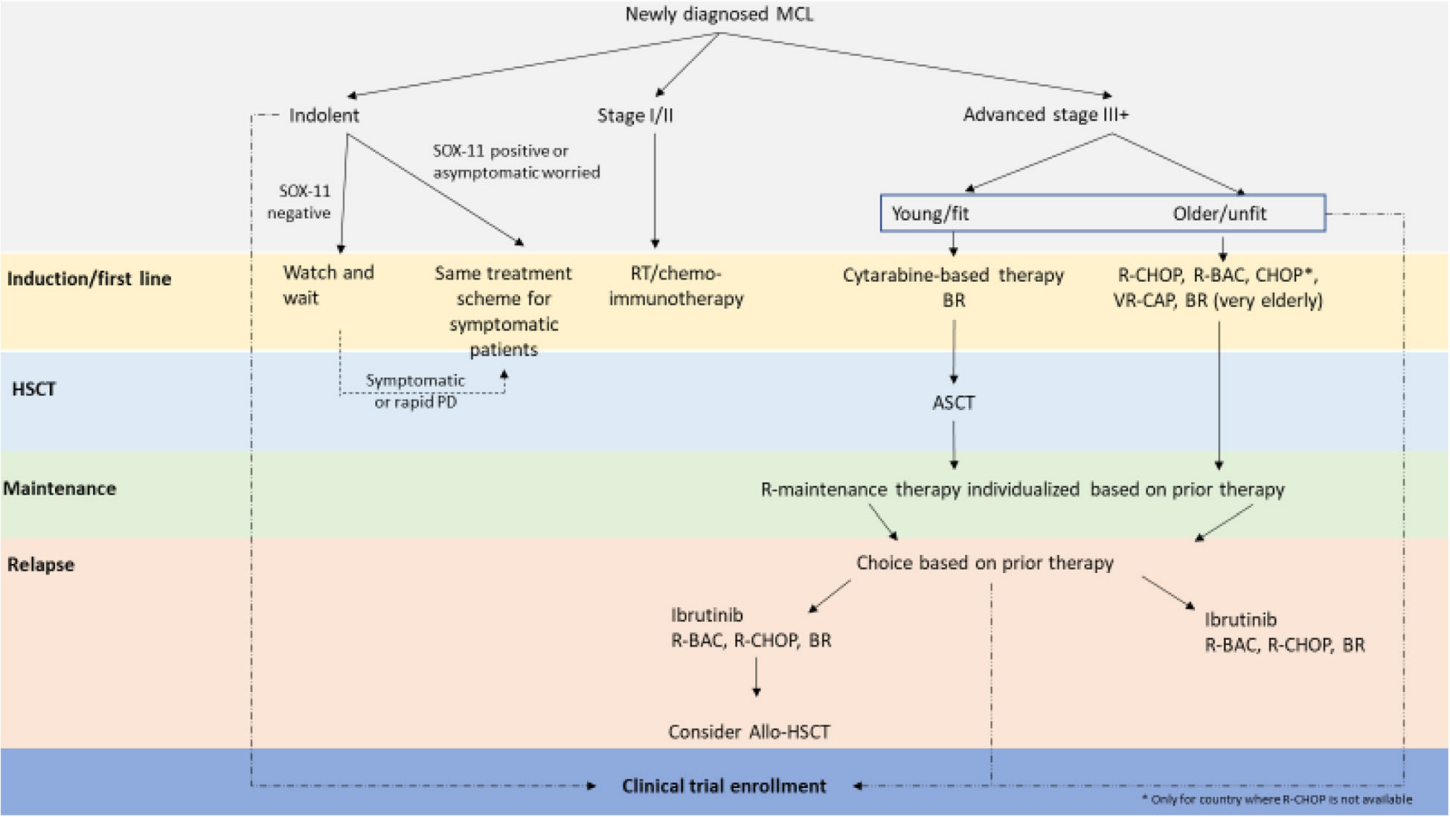
Flowchart of management of mantle cell lymphoma in Asian parents

With this in mind, the ALSG members considered available international guidelines and existing clinical data from Asia to formulate recommendations for the treatment of MCL in Asia as summarized in Table 3 and Fig. 1. In addition to this, specifically used regimens in each country in Asia are summarized in the supplementary appendix. These recommendations consider the current treatment landscape within Asia, including the availability of newer treatments and emphasize data-specific to the Asian population where available.

**Table 3:** Summary of ALSG recommendations

First-line treatment	
Indolent MCL	
• Adoption of a management strategy similar to CLL, utilizing a “watch-and-wait” approach, may be appropriate for asymptomatic patients with MCL. Typical clinical presentation of indolent disease comprises leukemic non-nodal CLL-like, including low tumor burden and Ki67 proliferation fraction < 10%. It is useful to confirm SOX11 negativity with hypermutated *IGHV* to determine clearly indolent disease. • Patients are often reluctant to undertake a “watch-and-wait” strategy. For asymptomatic patients desiring treatment, the same treatment scheme for symptomatic patients requiring treatment is considered. • Communication between the clinician and the patient, as well as the patients’ family members or caregivers, in the decision-making process is recommended. • Clinical trial enrollment is strongly suggested where possible.	
Stage I/II limited, non-bulky disease	
• Following ESMO and NCCN guidelines is appropriate for Asian MCL patients. • However, consideration could also be made to treat according to guideline recommendations for advanced disease, particularly if there are adverse histological features.	
Advanced stage: young, fit patients	
• Current practice for induction therapy in young fit patients differs across Asia based on drug availability and reimbursement status. • Few physicians use HyperCVAD; however, this risks omitting or reducing the dose of cytarabine, which may be the most useful drug. • Cytarabine has an important role as induction therapy in MCL but there is a lack of data supporting the best cytarabine dosage and dosing interval. • The interaction between cytarabine and purine analogs should be considered to address concerns around cytarabine dose and toxicity. • Clinical trial enrollment is strongly suggested where possible.	
Advanced stage: elderly, unfit patients	
• Choice of therapy in Asia is limited by drug availability and reimbursement status. • R-CHOP, R-BAC, CHOP, and VR-CAP are used in this setting in Asia. BR is the preferred regimen in elderly, unfit patients. • Bendamustine is associated with lower CD4 counts and an increased risk of infection. These risks must be monitored closely when considering this agent and prophylaxis for pneumocystis pneumonia should be considered. • Clinical trial enrollment is strongly suggested where possible.	
Role of HSCT	
• Some ALSG members felt that the high chance of relapse within 3–4 years, even with consolidation therapy, may justify ASCT in young fit patients. While the recommendation that all responding patients should receive ASCT has become generally accepted, this remains uncertain, especially with novel agents changing the therapeutic landscape. Therefore, for young women of reproductive age, ASCT should be avoided and in relatively older individuals for whom reproduction is not a concern, ASCT remains an important option. • In most Asian countries, HSCT is reimbursed by payors, whereas novel therapies are not; the decision for transplant may be financially driven rather than data-driven. However, real-world data may demonstrate the role or benefit of auto-SCT in some patients. • Whether high-dose therapy followed by ASCT can be safely omitted from intensive first-line therapy that incorporates a BTK inhibitor will be tested in a European clinical trial.	
Maintenance therapy after first-line treatment	
• The clinical benefit of rituximab-maintenance therapy following BR and VR-CAP is not convincing. • Rituximab-maintenance therapy should be recommended after R-CHOP or cytarabine-containing induction therapy. • The choice of rituximab-maintenance therapy in Asian patients should be individualized, and affordability and reimbursement status remain important considerations in the region.	
Relapsed/refractory treatment	
• Ibrutinib should be considered in the second rather than later-line setting. • Ibrutinib is tolerable in Asian patients. Adverse events (e.g., non-specific musculoskeletal symptoms, skin dryness or itching, changes to nails and hair) are not well characterized in the literature, and more Asian real-world data might be needed. • Follow-on BTK inhibitors including zanubrutinib could be considered if available, but a difference to ibrutinib in terms of efficacy and safety remains to be shown. • Clinical trial enrollment is strongly suggested where possible, especially for patients with TP53 mutation associated with poor prognosis.

Our work also highlights several remaining unmet needs and required future research directions in Asia. Limited data on the epidemiology of MCL within Asian populations were found, emphasizing the importance of comprehensive and contemporary registry data. It is recognized that ethnic characteristics can affect treatment efficacy and side effect profiles [81]. With the availability of promising therapies for MCL based predominantly on Western datasets, this further emphasizes the importance of additional clinical trial data within Asian populations to better inform the appropriate use of MCL treatments in this population. Finally, this is the first consensus the ALSG made for MCL. There is no clear “one-size-fits-all” standard therapy in MCL considering the heterogeneity of the disease and variability in the availability of treatments by country/region. In addition, the various treatment sequence approaches are an unanswered question for this disease that remains, at this point, widely accepted to be incurable.

## Conclusion

In conclusion, this paper reviewed available treatment for MCL, and where clinical gaps were identified, provided expert consensus from the ALSG on appropriate MCL management in Asia. Accordingly, it was found that Asian patient-specific data for the treatment of MCL are lacking, and the availability of treatment options differs between country and region within Asia. Therefore, no clear one-size-fits-all approach exists and further investigation on the most appropriate sequence of treatment should be considered for this heterogeneous disease.

## Supplementary information

**Additional file 1: Supplementary Appendix.** Specifically used regimens in Asia

## Data Availability

Not applicable

## Abbreviations

Allo-HSCT: Allogeneic hematopoietic stem cell transplantation
ALSG: Asian Lymphoma Study Group
Ara-C: Cytarabine
Auto-HSCT: Autologous hematopoietic stem cell transplantation
BR: Bendamustine, rituximab
BSH: British Society of Haematology
BTK: Bruton’s tyrosine kinase
CLL: Chronic lymphocytic leukemia
CR: Complete response
CT: Computed tomography
EFS: Event-free survival
ESMO: European Society of Medical Oncology
HR: Hazard ratio
HSCT: Hematopoietic stem cell transplantation
HyperCVAD: Hyperfractionated cyclophosphamide, vincristine, doxorubicin, dexamethasone, alternating with high-dose methotrexate and cytarabine
IGHV: Immunoglobulin heavy chain variable region
LEF1: Lymphoid enhancer-binding factor 1
MCL: Mantle cell lymphoma
MIPI: Mantle Cell Lymphoma International Prognostic Index
NCCN: National Comprehensive Cancer Network
NHL: Non-Hodgkin lymphoma
ORR: Objective response rate
OS: Overall survival
PET: Positron emission tomography
PFS: Progression-free survival
R-BAC: Rituximab, cytarabine, bendamustine
R-CHOP: Rituximab, cyclophosphamide, doxorubicin, vincristine, prednisone
R-DHAP: Rituximab, dexamethasone, cytarabine, cisplatin
R-FC: Rituximab, fludarabine, cyclophosphamide
R-HyperCVAD: Rituximab, hyperfractionated cyclophosphamide, vincristine, doxorubicin, dexamethasone, alternating with high-dose methotrexate and cytarabine
VR-CAP: Bortezomib, rituximab, cyclophosphamide, doxorubicin, prednisone
