# Supplementary material for: Treatment of mantle cell lymphoma in Asia: a consensus paper from the Asian Lymphoma Study Group
Source: J Hematol Oncol. 2020 Mar 17;13:21. doi: 10.1186/s13045-020-00855-9 (PMC7079508; doi:10.1186/s13045-020-00855-9)
Supplement: Supplementary file 1 — Additional file 1: Supplementary Appendix. Specifically used regimens in Asia [file 13045_2020_855_MOESM1_ESM.pdf]

## Supplementary Appendix

**Table.** Specifically used regimens in Asia

| **Location** | **Induction therapy in young, fit patients** | **Induction therapy in older, unfit patients** | **Maintenance therapy** | **Relapsed/ refractory MCL 2nd-line** | **Relapsed/ refractory MCL later line** |
|---|---|---|---|---|---|
| China | Alternating R-CHOP and R-DHAP followed by auto-HSCT | R-CHOP or lenalidomide | CAR-T used in clinical trial setting | Ibrutinib | Lenalidomide |
| Hong Kong | R-hyperCVAD or R-BAC; auto-HSCT | R-BAC if patient cannot tolerate ara-C; BR in very old, unfit patients; IR if patient can afford | Rituximab used in some patients | Ibruitinib | Bendamustine, ara-C or venetoclax |
| Taiwan | Hyper-CVAD or high-dose ara-C or CHOP (rituximab not reimbursed) followed by auto-HSCT | CHOP or CVP, VR-CAP may be an option | N/A | Ara-C | Ara-C |
| Japan | R-CHOP followed by CHASER then auto-HSCT | BR (mostly) or VR-CAP (less common) | Rituximab recommended per guidelines | Ibrutinib | Bendamustine, CHOP, or R-CHOP |
| Korea | R-CHOP followed by high-dose ara-C then HSCT; BR preferred but not reimbursed | VR-CAP or R-CHOP followed by high-dose ara-C; BR preferred but not reimbursed | Rituximab used in some patients | Ibrutinib | Bortezomib, lenalidomide or ara-C |

Ara-C, cytarabine; Auto-HSCT, autologous hematopoietic stem cell transplantation; BR, bendamustine, rituximab; CAR-T, chimeric antigen receptor T-cell; CHOP, cyclophosphamide, doxorubicin, vincristine, prednisone; CVP, cyclophosphamide, vincristine, prednisone; MCL, mantle cell lymphoma; R-BAC, rituximab, cytarabine, bendamustine; R-CHOP, rituximab, cyclophosphamide, doxorubicin, vincristine, prednisone; R-DHAP, rituximab, dexamethasone, cytarabine, cisplatin;

R-HyperCVAD, rituximab, hyperfractionated cyclophosphamide, vincristine, doxorubicin, dexamentasone, alternating with high-dose methotrexate and cytarabine; VR-CAP, bortezomib, rituximab, cyclophosphamide, doxorubicin, prednisone.
